# Multimodal data fusion: integrating PET/MRI and liquid biopsy for a holistic view of cancer biology

**DOI:** 10.3389/fonc.2026.1775271

**Published:** 2026-05-08

**Authors:** Yuying Zhao, Bo Shen, Wei Luo, Mingli Wang

**Affiliations:** Department of Radiology, Huzhou Central Hospital, Huzhou, Zhejiang, China

**Keywords:** CtDNA, liquid biopsy, multimodal data fusion, PET/MRI, precision medicine

## Abstract

The systemic complexity and spatiotemporal heterogeneity of cancer demand diagnostic strategies beyond single modalities. The integration of positron emission tomography/magnetic resonance imaging (PET/MRI) with liquid biopsy offers a revolutionary paradigm for a comprehensive view of tumor biology. This approach synergizes PET/MRI’s high-resolution spatial information—covering anatomical, functional, and metabolic characteristics—with the systemic, dynamic molecular data from liquid biopsy, particularly circulating tumor DNA (ctDNA). This review systematically examines the principles of this synergy, analyzing multimodal data fusion strategies, clinical evidence, and future challenges. The central benefit of this fusion is the enhancement of clinical decision-making across the cancer care continuum: improving early diagnosis and localization, resolving spatial heterogeneity, enabling dynamic monitoring of treatment efficacy, and tracing drug resistance. Current evidence, though primarily from retrospective or proof-of-concept studies, strongly supports this potential. However, significant challenges persist in technical standardization, algorithmic development for integrating heterogeneous data, and the need for large-scale prospective validation. Propelled by advances in artificial intelligence, overcoming these hurdles will shift oncology from static diagnostics toward a new era of precision medicine, capable of dynamically mapping the entire tumor ecosystem to deliver truly individualized patient care.

## 1 Introduction

Cancer, one of the leading causes of death worldwide, is fundamentally a complex systemic disease driven by genomic instability and characterized by dysregulation across multiple layers of biological networks (1–3). Despite significant advances in surgery, radiotherapy, chemotherapy, targeted therapy, and immunotherapy, tumor heterogeneity, dynamic microenvironmental adaptations, and therapeutic resistance remain the core challenges contributing to clinical failure (4). Traditional diagnostic paradigms, such as histopathology-based “gold standards” or single-modality imaging, often provide only a static snapshot of a localized region at a single time point, failing to capture the comprehensive spatiotemporal evolution of tumors (5, 6).

Against this backdrop, multimodal data fusion has emerged as a critical strategy for deciphering the complexity of cancer. This strategy aims to integrate complementary information from diverse technological platforms to construct a more complete and dynamic model of the disease. Among these, the integration of macroscopic imaging technologies with microscopic molecular assays is particularly compelling. positron emission tomography/magnetic resonance imaging (PET/MRI), as an advanced hybrid imaging technology, enables the simultaneous acquisition of anatomical details with high soft-tissue contrast, functional parameters (such as perfusion and diffusion), and specific molecular metabolic information (7, 8). Simultaneously, liquid biopsy, by analyzing circulating tumor DNA (ctDNA), circulating tumor cells (CTCs), exosomes, and other components in peripheral blood, enables non-invasive, real-time, and longitudinal monitoring of tumor burden, genetic mutation profiles, clonal evolution, and the immune microenvironment (9, 10).

However, reliance on any single technology carries inherent limitations. While PET/MRI excels at precisely localizing lesions and delineating their local microenvironment, it has limited sensitivity for detecting micrometastases (<5 mm) and cannot reveal the underlying molecular mechanisms driving tumor evolution (11, 12). Conversely, while liquid biopsy can sensitively capture systemic molecular signals, it lacks critical spatial localization information, making it difficult to distinguish contributions from primary versus metastatic sites or to directly assess local microenvironmental conditions (13–15). Therefore, the deep integration of PET/MRI’s “spatial-functional” advantages with the “temporal-molecular” strengths of liquid biopsy has the potential to create a powerful synergistic effect, transcending the limitations of either approach alone.

Currently, this integrated concept has demonstrated significant potential across multiple cancer types, yet it continues to face numerous challenges. First, how can standardized data acquisition and processing protocols be established to ensure the comparability and reliability of multi-source data? Second, how can we develop efficient computational models to integrate high-dimensional, heterogeneous radiomics and genomics data? Ultimately, can this integrated strategy be prospectively validated in clinical trials to improve patient outcomes? This article systematically reviews the biological foundations and technological frontiers of PET/MRI and liquid biopsy, provides an in-depth analysis of their complementary rationale and integrative mechanisms, and summarizes existing clinical evidence, aiming to establish a comprehensive theoretical and practical framework for advancing multimodal precision oncology.

## 2 PET/MRI and liquid biopsy: technical principles, clinical value, and inherent limitations

### 2.1 PET/MRI: an integrated imaging platform for multidimensional information

By integrating the functional metabolic imaging capabilities of PET with the superior soft-tissue resolution and multiparametric functional imaging of MRI within a single device, PET/MRI enables multiscale and multidimensional characterization of tumor biological behavior (16). The MRI component not only provides detailed anatomical structures but also utilizes diffusion-weighted imaging (DWI) to quantify the Brownian motion of water molecules, reflecting cellular density; employs dynamic contrast-enhanced (DCE-MRI) to assess vascular permeability and perfusion; and leverages magnetic resonance spectroscopy (MRS) to non-invasively detect the concentrations of specific metabolites (17, 18). The PET component involves injecting radiolabeled tracers (such as ^18^F-FDG, ^68^Ga-PSMA, ^18^F-FET, etc.) to target key biological processes like glucose metabolism, specific receptor expression, or amino acid transport, thereby revealing the molecular phenotype of the tumor (19–21). The development of next-generation PET tracers continues to expand imaging capabilities; for example, Huang et al. recently reported ^64^Cu-NOTA-Ivonescimab, a novel ImmunoPET tracer targeting VEGF-A in colorectal carcinoma, demonstrating specific tumor uptake of 13.73 ± 0.95%ID/g at 48 hours post-injection (22).

In clinical practice, PET/MRI has demonstrated superior value over positron emission tomography/computed tomography (PET/CT) in the diagnosis, staging, and treatment response assessment of soft-tissue occupying lesions, including brain tumors, prostate cancer, gynecological malignancies, and hepatobiliary-pancreatic tumors (23–26). For instance, in glioblastoma, ^18^F-FET PET/MRI can effectively differentiate between tumor recurrence and radiation necrosis, with diagnostic accuracy significantly surpassing that of MRI alone (27). In prostate cancer, ^68^Ga-PSMA PET/MRI has emerged as the preferred method for localizing lesions following biochemical recurrence, profoundly transforming clinical decision-making pathways (28). However, the inherent limitations of PET/MRI cannot be overlooked. While the MRI component provides high structural resolution, the overall system’s ability to characterize small lesions (<5 mm) is often constrained by the physical spatial resolution of the PET component. This limitation primarily affects metabolic sensitivity and quantification, potentially leading to the underestimation of activity in micrometastases due to partial volume effects (29). Additionally, tracers may exhibit non-specific uptake, leading to false-positive results. More importantly, PET/MRI is inherently a local imaging technique, with its field of view confined to the scanned area. This makes it difficult to comprehensively assess systemic tumor burden and overall biological status, creating a blind spot in understanding the full evolutionary trajectory of the cancer.

### 2.2 Liquid biopsy: a molecular window for non-invasive dynamic monitoring

**Figure 1:**
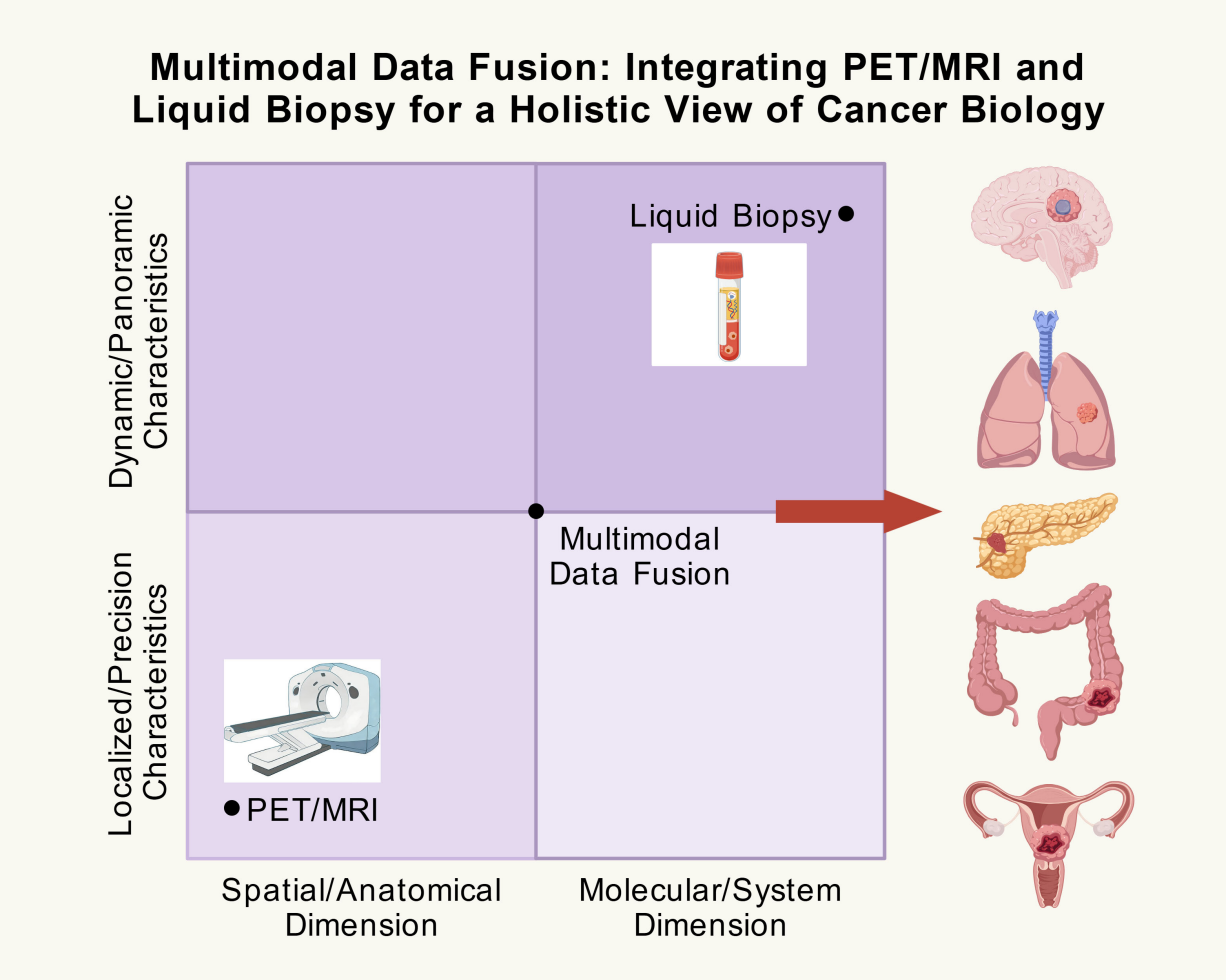
Diverse biofluid sources and key analytes in liquid biopsy. Liquid biopsy enables non-invasive tumor monitoring by analyzing tumor-derived materials in various bodily fluids. As illustrated, samples can be obtained from peripheral blood, urine, saliva, and cerebrospinal fluid (CSF). The central panel highlights the key molecular analytes detectable in these fluids: circulating tumor DNA (ctDNA) carrying genetic mutations; microRNA (miRNA) reflecting regulatory changes; extracellular vesicles (EVs) mediating intercellular communication; and circulating tumor cells (CTCs) shed from primary or metastatic lesions. This multi-source, multi-analyte approach provides a comprehensive molecular snapshot of the disease.

Liquid biopsy refers to the non-invasive monitoring of tumors by analyzing tumor-derived materials in body fluids such as blood, urine, saliva, and cerebrospinal fluid (CSF). Its core components include ctDNA, CTCs, and exosomes (9, 30, 31) (**Figure 1**). ctDNA is fragmented DNA released into the bloodstream from apoptotic or necrotic tumor cells, carrying somatic mutations, copy number variations, and methylation profiles highly consistent with those of the primary tumor. It is currently the most widely used liquid biopsy biomarker (32, 33). Multiple studies have demonstrated that ctDNA levels are significantly correlated with tumor stage, burden, and prognosis, and their dynamic changes can predict treatment response or resistance weeks to months earlier than imaging modalities (34). For example, in non-small cell lung cancer (NSCLC), the clearance of epidermal growth factor receptor (EGFR)-mutant ctDNA is a powerful predictor of long-term survival in patients undergoing EGFR-Tyrosine Kinase Inhibitor (TKI) therapy (35). Recent advances have further refined ctDNA quantification methodologies; for instance, Ryu et al. developed a methylation-based approach that achieved 82% sensitivity and 93% specificity for colon cancer detection by quantifying ctDNA among cell-free DNA using cancer-specific hypermethylated regions (36).

CTCs are intact cancer cells shed into the bloodstream from primary or metastatic lesions. They retain cellular morphology, protein expression, and proliferative potential, enabling applications such as single-cell sequencing, *in vitro* culture, and drug sensitivity testing, thereby providing a valuable resource for studying tumor metastasis mechanisms (37). Exosomes are nano-sized vesicles secreted by cells, containing bioactive molecules such as proteins and nucleic acids (e.g., microRNA (miRNA), lncRNA). They play a crucial role in mediating tumor-microenvironment communication, immune evasion, and distant organ colonization (38–40). Despite its promising prospects, the clinical application of liquid biopsy still faces significant challenges. In patients with early-stage or low-tumor-burden cancer, ctDNA concentrations are extremely low (often below 0.1% mutant allele frequency), placing exceptionally high demands on the sensitivity of detection technologies (41). Standardization across different detection platforms, such as droplet digital polymerase chain reaction (ddPCR) and next-generation sequencing (NGS), has not yet been unified, raising concerns about the comparability of results (42). Most critically, while liquid biopsy provides a panoramic perspective, it entirely lacks spatial localization information of lesions, rendering it unable to answer the essential question of “which lesion the mutation originates from”.

**Figure 2:**
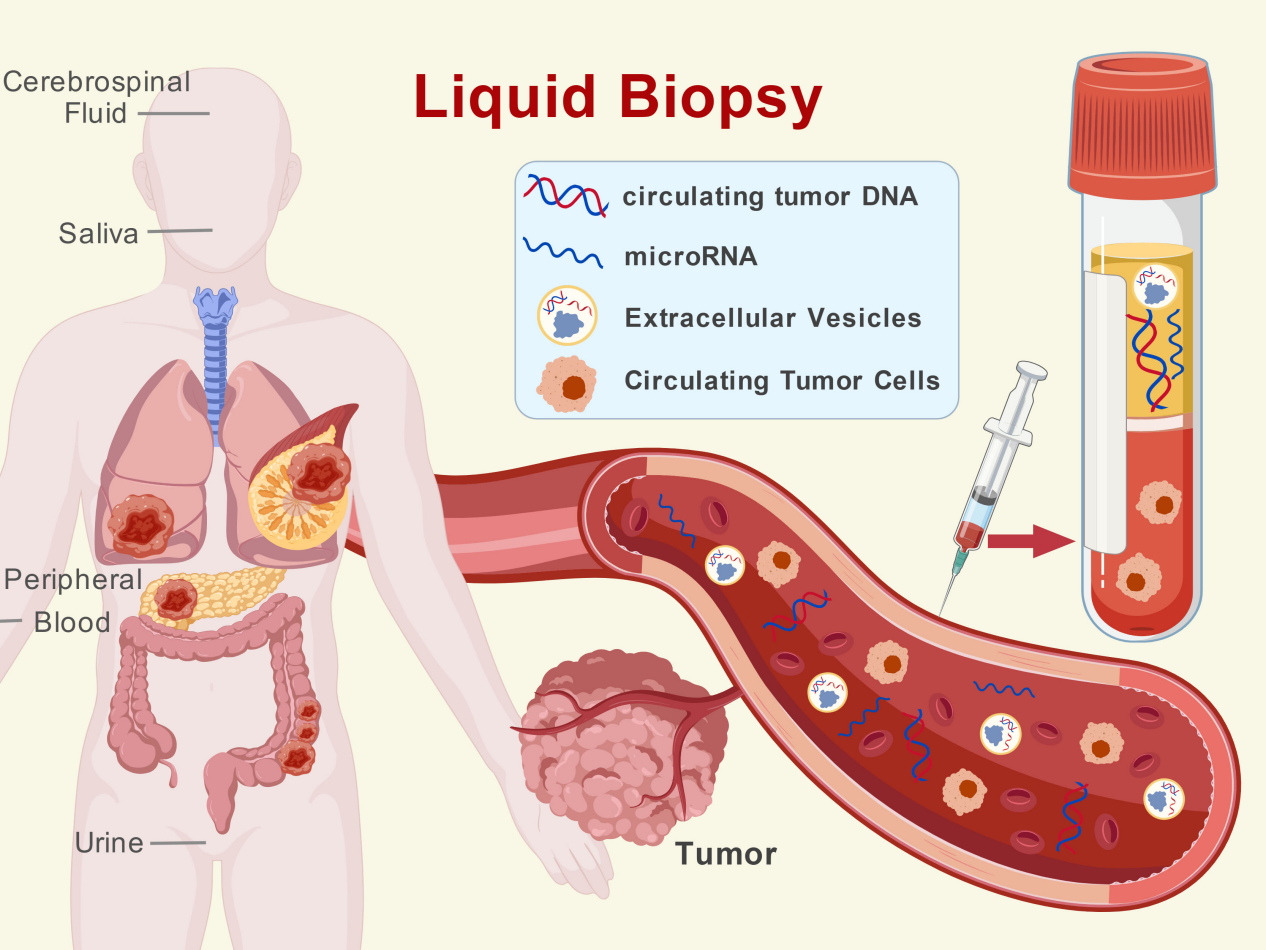
Schematic representation of complementary dimensions between PET/MRI and liquid biopsy. This schematic illustrates how PET/MRI and liquid biopsy compensate for each other’s inherent limitations. PET/MRI (left) excels in providing high-resolution spatial imaging, multiparametric functional data, and local microenvironment characterization (e.g., perfusion, cellularity), but it is inherently localized. In contrast, liquid biopsy (right) offers non-invasive, systemic monitoring with high temporal resolution and comprehensive molecular profiling (including ctDNA, CTCs, EVs, and miRNA), providing early predictive capabilities that imaging may miss. Their integration bridges the gap between local-spatial phenotype and systemic-temporal genotype.

As illustrated in **Figure 2**, PET/MRI and liquid biopsy exhibit inherent complementarity across multiple dimensions, including space-time, local-systemic, and structure-molecular axes. PET/MRI provides high-resolution spatial imaging and multi-functional parameters, excelling in the characterization of the local microenvironment; whereas liquid biopsy enables non-invasive, systemic, and high-temporal-resolution molecular dynamic monitoring through biomarkers such as ctDNA, CTCs, and exosomes. This complementarity and synergy of multidimensional information lay the technical foundation for transcending the diagnostic limitations of single-modality approaches.

### 2.3 Clinical significance: moving beyond the diagnostic and therapeutic dilemmas of single-modal approaches

The aforementioned technical limitations directly contribute to numerous challenges in clinical practice. To systematically illustrate these opposing yet complementary characteristics, **Table 1** provides a detailed, side-by-side comparison of PET/MRI and liquid biopsy across key technical dimensions, including their typical turnaround times (PET/MRI: 1–2 days; liquid biopsy: 3–14 days depending on assay complexity) (43, 44). For instance, in the era of immunotherapy, imaging assessments are often misinterpreted due to “pseudoprogression”, while a reduction in ctDNA alone may result from the control of non-target lesions and does not necessarily reflect the overall disease status (45). When assessing tumor heterogeneity, a single tissue biopsy only represents the clonal composition at the sampled site and can easily miss key drug-resistant subclones. In contrast, while liquid biopsy can integrate information from all clones, it cannot reveal the spatial distribution patterns of these clones (46, 47). These challenges collectively point to a solution: the integration of spatially resolved imaging information with temporally resolved molecular data. Such integration not only enhances diagnostic accuracy but also provides unprecedented insights into understanding the tumor ecosystem, predicting treatment response, and guiding personalized interventions, thereby truly advancing the paradigm shift from “empirical medicine” to “precision medicine”.

**Table 1:** Comparison of key technical characteristics of PET/MRI and liquid biopsy.

Dimension	PET/MRI	Liquid biopsy
Primary Information Type	Spatial, anatomical, functional, and metabolic phenotype. Quantitative metrics: SUVmax, SUVmean, MTV, TLG, ADC, DCE-MRI perfusion parameters	Systemic, molecular genotype and epigenotype. Quantitative metrics: ctDNA concentration (ng/mL), VAF (%), MAF, total alteration number
Spatial Resolution	Excellent. MRI: <1 mm; PET: ~3–4 mm (50)	None. Cannot provide any spatial localization information
Temporal Resolution	Low. Provides a static “snapshot.” Scans typically performed weeks or months apart.	High. Enables continuous monitoring through frequent sampling (days to weeks)
Detection Limit/Sensitivity	Limited by PET physical resolution: lesions <5 mm are challenging (29)	Advanced cancers: >90%; Stage I cancers: <50%. Can detect MAF as low as 0.1% with advanced NGS (41, 42)
Coverage Scope	Local to whole-body, but limited to the defined field of view per scan	Systemic. Captures molecular information from all tumor sites
Typical Turnaround Time	Fast. Scan: 1–2 hours; full report: typically 1–2 days (43)	Variable. Targeted assays (ddPCR): ~3–5 days; comprehensive NGS: ~7–14 days (44)
Invasiveness	Non-invasive (requires intravenous tracer injection)	Minimally invasive (simple peripheral blood draw)
Radiation Exposure	~6.6 mSv (mean) per scan (52)	None
Primary Clinical Applications	Lesion localization, staging, local treatment response assessment, surgical/radiotherapy planning	Early detection, MRD monitoring, clonal evolution tracking, resistance mutation detection
Key Limitations	Insensitive to micrometastases; high cost; radiation exposure	Lacks spatial context; low sensitivity in early-stage cancers; pre-analytical variability; lack of standardization

### 2.4 Unique synergistic advantages over alternative multimodal approaches

While various multimodal strategies exist, the specific pairing of PET/MRI with liquid biopsy offers a unique synergy that addresses the limitations of other combinations. A critical comparison clarifies its distinct value:

Superiority over PET/CT + liquid biopsy: While PET/CT is a powerful tool, PET/MRI offers three key advantages in the context of fusion with liquid biopsy. First, MRI provides vastly superior soft-tissue contrast, enabling more precise localization and characterization of lesions in organs like the brain, prostate, liver, and female reproductive system (48–50). This is crucial for accurately mapping a systemic molecular signal (from ctDNA) to a specific anatomical lesion. Second, MRI’s multiparametric capabilities (e.g., DWI, DCE-MRI, MRS) provide deep functional and metabolic phenotyping, offering richer data on cellularity, perfusion, and the microenvironment. This creates a more detailed “phenotypic anchor” for the molecular data than the primarily anatomical information from CT. Third, and critically for a strategy centered on dynamic monitoring, PET/MRI significantly reduces the patient’s cumulative radiation dose (mean effective dose ~6.6 mSv) compared to repeated PET/CT scans, making it far more suitable for the longitudinal follow-up paradigms enabled by liquid biopsy (51, 52).

Complementarity to MRI + Tissue Genomics: The traditional gold standard of pairing imaging with tissue genomics is limited by the invasive nature and spatiotemporal constraints of tissue biopsies. A tissue sample represents a single geographical point at a single moment, suffering from sampling bias and often failing to capture the full spectrum of intra-tumor heterogeneity and clonal evolution (53). Liquid biopsy overcomes this by providing a minimally invasive, systemic, and longitudinal view of the tumor’s entire molecular landscape. It captures the “genomic average” of all lesions and allows for continuous monitoring of molecular changes over time, offering a dynamic perspective that tissue genomics cannot match.

Advancement beyond Radiomics-only Models: Radiomics models aim to infer underlying tumor biology and genetic status from quantitative imaging features alone (54). While powerful, these models are correlational and can lack biological interpretability. The integration of liquid biopsy provides direct, causal molecular “ground truth” (e.g., specific driver mutations, methylation status). This allows for the development of more robust and biologically plausible fusion models that explicitly link the imaging phenotype with the molecular genotype, moving beyond prediction to a deeper biological understanding and enhancing the reliability of the model.

## 3 Strategies and collaborative mechanisms for multimodal data fusion

### 3.1 Data fusion strategies

Early Fusion (Data-Level Fusion) directly inputs raw imaging data and molecular data into the model, which can maximize the retention of information. However, this approach places extremely high demands on the algorithm’s ability to process high-dimensional heterogeneous data (55). For example, developing a single neural network that can simultaneously process raw volumetric imaging data (a 3D grid of voxels) and non-spatial genomic data (a 1D vector of mutation frequencies) at the input layer is non-trivial, as it requires highly specialized architectures to reconcile these fundamentally different data structures (56). Mid-level Fusion (Feature-Level Fusion) is currently the most prevalent and practical strategy. This approach involves separately extracting quantitative features from each modality before integrating them. For instance, a successful pipeline might begin by extracting a high-dimensional set of radiomic features from PET/MRI (e.g., using open-source libraries like PyRadiomics) that describe tumor shape (sphericity), intensity (SUVmax), and texture (heterogeneity, entropy). Concurrently, key molecular features are quantified from the liquid biopsy, such as ctDNA concentration, the variant allele frequency (VAF) of a specific driver mutation, or the total number of detected mutations. These distinct feature sets are then concatenated and fed into multivariate statistical or machine learning models (e.g., using established frameworks such as Scikit-learn, TensorFlow, or PyTorch) to explore associations and build predictive models (55, 57). A biologically plausible model might find that higher tumor metabolic heterogeneity on PET correlates with a greater number of mutations detected in ctDNA, together predicting a higher risk of treatment resistance. However, as these models increase in complexity, they often suffer from a ‘black box’ problem, making it difficult to understand their decision-making process. To address this critical challenge of model interpretability, techniques from the field of eXplainable AI (XAI) are becoming crucial. Methods such as SHAP (SHapley Additive exPlanations) can be applied *post-hoc* to quantify the contribution of each feature—both radiomic and genomic—to a specific prediction, enhancing transparency and clinical trust. Late Fusion (Decision-Level Fusion) allows the two modalities to independently analyze and draw preliminary conclusions (such as imaging progression or molecular drug resistance). These conclusions are then integrated through rule-based methods for a comprehensive judgment. While this approach offers flexibility, it may result in the loss of deep interactive information between the modalities (58).

### 3.2 Complementary synergy across spatial-temporal dimensions

The integration of PET/MRI and liquid biopsy is fundamentally rooted in their intrinsic complementarity across information dimensions. PET/MRI provides high spatial resolution for the localized characterization of anatomical structures, functional parameters, and metabolic states, enabling precise profiling of tumors and their microenvironments at specific time points. In contrast, liquid biopsy facilitates high temporal resolution systemic monitoring, allowing continuous tracking of the dynamic evolution of tumor molecular burden and genomic characteristics throughout the body. This combination of spatial localization capabilities and temporal sequence-based dynamic monitoring enables researchers to simultaneously capture the morphological and functional characteristics of lesions alongside their underlying molecular biological trajectories (59, 60).

### 3.3 Information fusion across local-systemic levels

From the perspective of information coverage, PET/MRI technology primarily targets localized anatomical regions. Its advantage lies in the precise delineation of interactions between tumor tissue and its local microenvironment, including but not limited to spatial heterogeneity features such as neovascular density, hypoxic area distribution, and immune cell infiltration. Complementing this, liquid biopsy reflects systemic molecular status, integrating detection signals that combine molecular information from primary lesions, metastatic sites, and potential micrometastases (61–63). The integration of these two technological approaches theoretically enables the construction of a comprehensive analytical framework spanning from localized fine-scale characterization to systemic overall assessment. For example, when PET/MRI detects a lesion area exhibiting high metabolic activity and hyperperfusion characteristics, and simultaneously, liquid biopsy shows a significant increase in the abundance of the specific driver gene mutations corresponding to that lesion in ctDNA, this multimodal evidence combination not only confirms the clinical significance of the lesion as the primary source of tumor burden but also suggests its crucial biological value as a potential site for the advantageous growth of resistant clones (30, 64, 65).

### 3.4 Structural-molecular cross-validation

In terms of information types, PET/MRI primarily provides indirect functional correlation information based on biophysical characteristics. For example, an increase in the Standardized Uptake Value (SUVmax) may indicate enhanced glucose metabolic activity in the tumor region, while changes in the Apparent Diffusion Coefficient (ADC) on DWI can reflect alterations in tissue cellular density. These parameters characterize the functional imaging features exhibited by tumors under specific pathophysiological conditions, representing correlation information at the “phenotypic-functional” level (66, 67). In contrast, liquid biopsy can provide more direct molecular mechanism information with stronger causal implications. For example, detecting specific mutations in the Kirsten Rat Sarcoma Viral Oncogene Homolog (KRAS) gene in ctDNA can directly confirm the abnormal activation state of the Mitogen-Activated Protein Kinase (MAPK) signaling pathway, while analyzing the expression profiles of specific miRNAs in exosomes can reveal regulatory networks between tumor cells and the microenvironment. Integrating and cross-validating these two types of information can establish a more reliable framework for biological inference. Specifically, when PET imaging using radiolabeled Programmed Death-Ligand 1 (PD-L1) inhibitors shows increased tracer uptake in a specific region, and simultaneously, significantly elevated levels of PD-L1 protein expression or PD-L1 mRNA abundance are detected in CTCs or tumor-derived exosomes from samples collected during the same period, this provides support through multiple independent lines of evidence for the immunosuppressive state of the tumor microenvironment in that region. Such mutual corroboration of multimodal data significantly enhances the biological reliability and clinical translational value of findings derived from any single detection method (68, 69).

### 3.5 Powerful support from preclinical studies

Multiple preclinical studies have provided a solid theoretical foundation for this integrative strategy. For instance, in colorectal and lung cancers, individuals with positive screening results based on ctDNA methylation have achieved early lesion localization through PET/MRI (59, 70). In the efficacy evaluation of lymphoma and breast cancer, the combined analysis of early treatment PET/MRI parameters and ctDNA dynamics has proven to be a powerful indicator for predicting progression-free survival (PFS) (71, 72). These findings, much like the early clinical trials of vascular endothelial growth factor (VEGF)/angiopoietin-2 (ANG2) bispecific antibodies, have demonstrated biological activity and tolerability, yet their efficacy as standalone strategies remains limited. Their greatest potential lies in their synergy with existing standard treatments, such as chemotherapy and immunotherapy (73).

## 4 Clinical application and translational prospects

**Figure 3:**
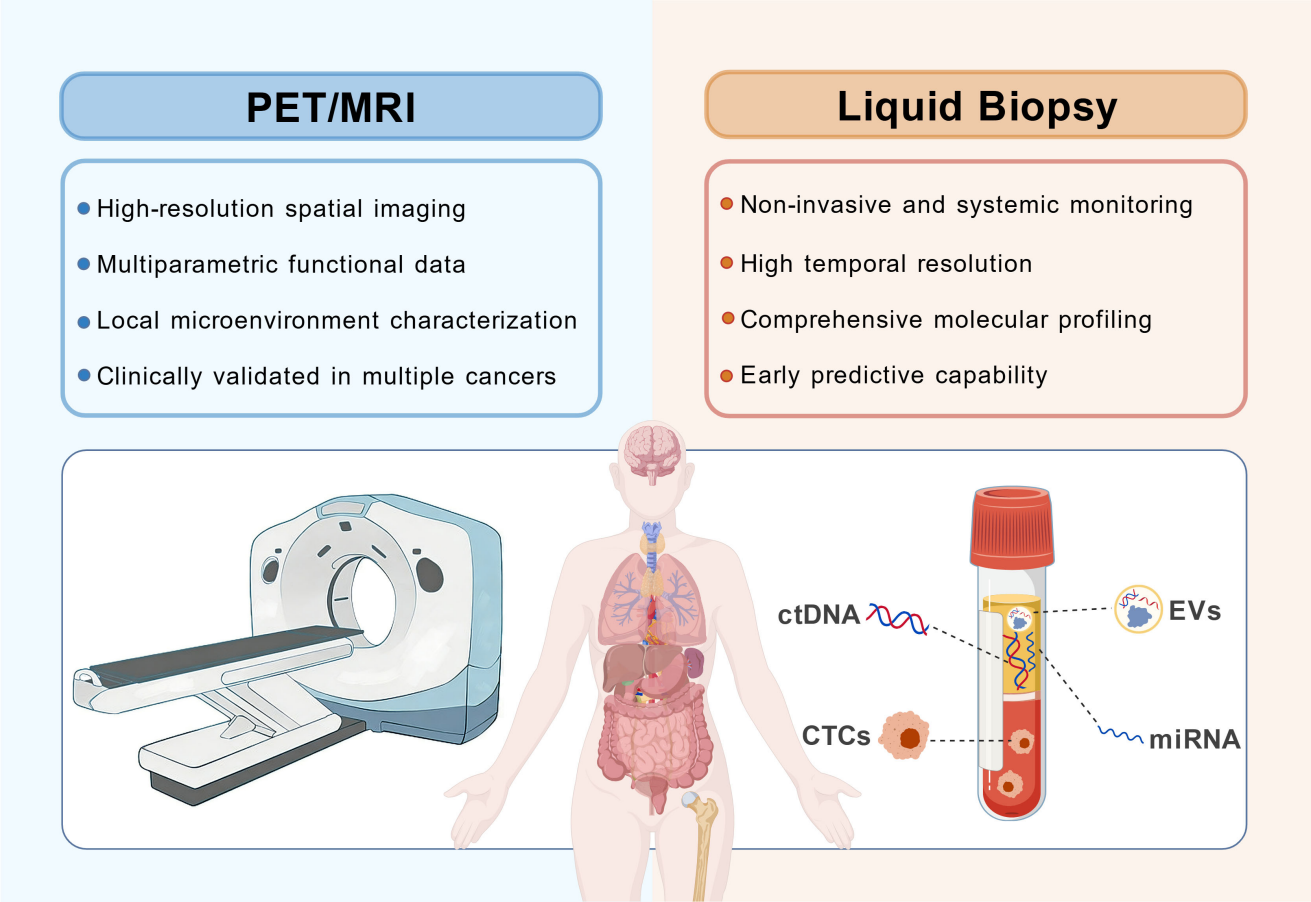
Clinical decision weight of PET/MRI and liquid biopsy across the cancer care continuum. The graph depicts the shifting clinical utility (decision weight) of PET/MRI (green line) and liquid biopsy (yellow line) during different phases of cancer management. Early Diagnosis: Liquid biopsy holds high weight for initial screening, while PET/MRI is crucial for precise lesion localization. Treatment Efficacy Evaluation: Both modalities contribute, with liquid biopsy capturing early molecular response. Drug Resistance Monitoring: Their synergy peaks here, as liquid biopsy detects new mutations (e.g., T790M) and PET/MRI identifies the specific anatomical sites of resistant clones. Recurrence Monitoring: Liquid biopsy serves as a sensitive first-line tool for molecular relapse, with PET/MRI providing anatomical confirmation and staging.

Based on the aforementioned multidimensional complementary mechanisms, the multimodal data fusion of PET/MRI and liquid biopsy demonstrates significant value throughout the entire clinical diagnosis and treatment process (**Figure 3**). From early diagnosis and risk stratification to treatment efficacy evaluation, drug resistance monitoring, and even long-term follow-up and recurrence early warning, the two modalities can contribute with differential decision weights at different stages, collectively establishing a dynamic and precise tumor management system.

### 4.1 Early diagnosis and risk stratification

In early cancer screening, liquid biopsy (such as multi-cancer detection based on ctDNA methylation signatures) can serve as efficient preliminary screening tools. However, a key challenge in general screening settings is that the low prevalence of cancer can limit the positive predictive value (PPV) of these tests, which, despite high specificity, leads to a risk of false-positive results (59). Whole-body PET/MRI technology provides crucial secondary verification and stratification capabilities. By integrating metabolic imaging (such as ^18^F-FDG PET) with high-resolution anatomical and functional MRI, this technology enables systemic evaluation of individuals with positive liquid biopsy results: accurately localizing suspicious lesions, distinguishing between benign and malignant conditions through multi-parameter analysis (e.g., SUVmax, ADC values), and offering anatomical guidance for subsequent interventions (74). This sequential “liquid biopsy-based initial screening followed by imaging-based precise screening” model has shown promise in early-phase and prospective cohort studies (e.g., the PATHFINDER study (59)) to improve the specificity and efficiency of screening. Recent large-scale prospective studies have further advanced this field: the K-DETEK study validated a multimodal ctDNA-based MCED test in 9, 057 asymptomatic individuals, demonstrating 70.8% sensitivity and 99.7% specificity (75). Additionally, integrating hotspot mutations with methylation and fragmentomic profiles has been shown to enhance early-stage cancer detection (76). However, most available evidence remains exploratory or derived from retrospective analyses; large-scale, prospective validation of this integrated strategy is still ongoing.

### 4.2 Treatment efficacy evaluation

During the treatment process, liquid biopsy can monitor in real time the disappearance of driver gene mutations or the emergence of new resistance mutations (such as EGFR T790M and Estrogen Receptor 1 (ESR1) mutations), while PET/MRI can simultaneously assess changes in the metabolic activity of lesions (77, 78). Dynamic combined analysis of PET/MRI and liquid biopsy has been shown in proof-of-concept and retrospective studies to help distinguish treatment response patterns, such as pseudoprogression versus true progression. For example, in melanoma immunotherapy cohorts, declining ctDNA levels with stable or transiently enlarging lesions suggested pseudoprogression (79, 80). Nevertheless, prospective validation in larger, multicenter trials is currently lacking, and the improvement in accuracy over single-modality assessment remains to be quantified.

### 4.3 Drug resistance monitoring

The evolution of tumor drug resistance is a dynamic and heterogeneous process that extends beyond the static perspective offered by single-site tissue biopsy (81). The integration of PET/MRI and liquid biopsy provides a systemic solution to this challenge. Liquid biopsy enables the capture of resistance-related molecular events weeks to months prior to radiological progression through its high temporal resolution monitoring. For instance, in NSCLC, the emergence of EGFR T790M-mutant ctDNA serves as an early marker of acquired resistance to EGFR-TKI therapy (82). Similarly, in breast cancer, ESR1 mutations are closely associated with resistance to endocrine treatment (83). However, the spatial distribution of resistance mutations determines therapeutic strategies. At this point, PET/MRI, with its high spatial resolution, is capable of localizing drug-resistant lesions with elevated metabolic activity. This can be achieved through the use of targeted radiotracers—such as probes labeled for PD-L1 or EGFR—to specifically image and locate particular drug-resistant clones (84). What is more critical is that the integration of multimodal data can effectively distinguish complex clinical phenotypes: during immunotherapy, if imaging indicates lesion enlargement while ctDNA levels continuously decline, it suggests pseudoprogression caused by immune cell infiltration, and the original treatment regimen should be continued; conversely, if ctDNA levels rise synchronously, it indicates true progression and resistance, necessitating a change in therapy. This integration therefore holds the theoretical potential to enable early detection and spatial resolution of resistance mechanisms, and could in principle guide subsequent clinical decisions: either comprehensively adjusting systemic treatment regimens based on mutation profiles or implementing localized intensification therapies for isolated resistant lesions. However, it must be emphasized that current evidence for this guiding capability remains largely proof-of-concept, derived primarily from retrospective or small prospective studies (e.g., detection of EGFR T790M resistance mutations in NSCLC (82)). Prospective trials are needed to validate whether this integrated approach improves clinical decision-making and patient outcomes compared with standard-of-care monitoring. Consequently, it drives a paradigm shift in clinical practice from passively responding to resistance toward proactively managing the evolution of resistance.

### 4.4 Long-term follow-up and recurrence monitoring

Long-term follow-up serves as a critical component of cancer management, aiming to enable timely intervention through early detection of recurrence or metastasis. The traditional follow-up model relies on periodic imaging examinations (such as CT) and tumor marker tests, both of which have limitations: imaging methods are often associated with radiation exposure and exhibit insufficient sensitivity for detecting minute lesions, while conventional tumor markers are constrained by their lack of specificity and sensitivity. In recent years, the integration of PET/MRI and liquid biopsy has provided a novel strategy for achieving more precise and personalized long-term follow-up. Their synergistic application typically follows the paradigm of “liquid biopsy as the first-line monitoring tool, with PET/MRI for confirmation”. Liquid biopsy can serve as a first-line monitoring tool, enabling early molecular-level detection of recurrence through regular (e.g., every 3–6 months) testing of ctDNA. The reappearance or elevation of ctDNA levels often precedes the visualization of lesions on imaging by several months, thereby facilitating the monitoring of molecular recurrence (85, 86). Upon a positive liquid biopsy result or the emergence of clinical symptoms, whole-body PET/MRI can be promptly initiated. Leveraging its high sensitivity and multi-parameter imaging capabilities, it enables precise localization of recurrent or metastatic lesions, differentiation between post-treatment changes and active tumor tissue, and assessment of lesion metabolic activity and local invasiveness. This provides critical anatomical and functional evidence for guiding salvage treatment decisions, such as re-operation, targeted radiation therapy, or adjustments to systemic treatment regimens. This integrated strategy significantly enhances the sensitivity and specificity of long-term follow-up, holding promise for optimizing recurrence monitoring protocols and improving patient prognosis. This integrated strategy has shown encouraging results in retrospective and observational studies (85, 86), suggesting improved sensitivity for detecting molecular relapse before radiographic evidence appears. However, prospective trials with predefined integration algorithms and clinical decision pathways are required to confirm whether this approach translates into improved long-term outcomes, such as overall survival (OS) or quality of life.

To provide a structured overview of the evidence supporting these clinical applications, **Table 2** summarizes several key studies that have explored the synergy between advanced imaging and liquid biopsy for cancer management.

**Table 2:** Representative clinical studies on PET/CT, PET/MRI, liquid biopsy, or their integration in oncology.

Study (First author, year)	Cancer type	Study design	Sample size	Modality	Key findings
Hofman MS et al., 2020 (proPSMA) (19)	Prostate	Prospective, randomized, multicenter	302	PET/CT (^68^Ga-PSMA)	PSMA PET-CT had superior accuracy vs conventional imaging (92% vs 65%; p<0.0001).
Schrag D et al., 2023 (PATHFINDER) (59)	Multiple (screening)	Prospective cohort	6,621	Liquid biopsy (ctDNA) + Imaging	Cancer signal detected in 1.4%; PPV was 38%. Demonstrated feasibility of MCED testing.
Ferraro DA et al., 2021 (61)	Prostate	Prospective, single-center	42	PET/MRI (^68^Ga-PSMA)	Patient-based accuracy for significant cancer was 90% (sensitivity 96%, specificity 81%).
Lee JH et al., 2017 (79)	Melanoma	Prospective cohort	76 (discovery) + 29 (validation)	ctDNA (anti-PD-1)	Undetectable ctDNA at baseline/on therapy predicted response (72-77% vs 6%) and prolonged PFS/OS.
Bola S et al., 2025 (57)	Head & Neck	Retrospective cohort	35	ctDNA + PET/CT	Liquid biopsy outperformed PET-CT (sensitivity 83% vs 67%; specificity 95% vs 42%).
Tokac RH et al., 2025 (72)	Breast	Retrospective, single-center	47	FDG-PET/CT + ctDNA	ctDNA detection correlated with higher WB-MTV (p=0.002) and WB-TLG (p=0.006). VAF correlated with SUVmax.

### 4.5 Future directions in artificial intelligence fusion

To fully unlock the clinical and scientific value of multimodal data, reliance on advanced computational methods is essential. Artificial intelligence technologies, particularly deep learning, provide a powerful analytical framework for integrating high-dimensional, heterogeneous PET/MRI radiomic features with liquid biopsy genomic data. By constructing specialized multimodal fusion neural network models, the system can automatically learn and analyze the complex nonlinear relationships between radiomic features (such as texture and morphology) and molecular biomarkers (such as specific gene mutations and methylation levels), thereby generating comprehensive evaluation models with significantly superior predictive performance compared to traditional unimodal methods (87).

However, a major bottleneck currently facing the deep learning-based fusion of PET/MRI and liquid biopsy is the strong dependence on large-scale, paired, and accurately labeled multimodal datasets. In most clinical studies, the number of patients with simultaneous PET/MRI and longitudinal liquid biopsy data remains limited, and high-quality expert annotations (e.g., lesion-level spatial–molecular correspondence) are even scarcer. This data scarcity severely constrains model training, increases the risk of overfitting, and limits cross-center generalizability. Addressing this challenge will require collaborative efforts to establish standardized data repositories, as well as the development of data-efficient learning strategies, including transfer learning, self-supervised learning, federated learning, and synthetic data generation. Without such advances, the translational potential of deep learning in this multimodal domain may remain constrained despite its theoretical promise.

### 4.6 Key challenges in clinical implementation

Transitioning the integrated PET/MRI and liquid biopsy strategy from a research concept to a routine clinical tool involves surmounting significant systemic challenges. These can be categorized into technological, logistical, and ethical-regulatory domains.

#### 4.6.1 Technological and standardization hurdles

The reproducibility and comparability of data across different institutions are paramount for large-scale validation and clinical adoption. This requires strict adherence to standardized protocols for both modalities.

For PET/MRI, consensus guidelines from professional bodies such as the European Association of Nuclear Medicine (EANM) and the Society of Nuclear Medicine and Molecular Imaging (SNMMI) provide a critical foundation (88, 89). Standardization must encompass not only scanner calibration and quality control but also specific acquisition parameters (e.g., radiotracer dose, uptake time, scan duration) and image reconstruction algorithms (e.g., Ordered Subset Expectation Maximization (OSEM) parameters, post-reconstruction filters). Harmonization initiatives, such as the Quantitative Imaging Biomarkers Alliance (QIBA), are essential for ensuring that quantitative metrics (e.g., SUV, ADC) are comparable across different scanner models and centers.

For liquid biopsy, the pre-analytical phase is a major source of variability. Expert consensus recommendations from organizations like the European Society for Medical Oncology (ESMO) and the American Society of Clinical Oncology (ASCO) emphasize the importance of standardizing pre-analytical variables (90). This includes the choice of blood collection tubes (e.g., Streck Cell-Free DNA BCT^®^ vs. standard Ethylenediaminetetraacetic acid (EDTA) tubes to prevent genomic DNA contamination), the time between blood draw and plasma processing, and centrifugation protocols. Furthermore, the analytical validation of sequencing platforms (e.g., ddPCR vs. NGS) must be harmonized, with clear reporting standards for sensitivity, specificity, and limits of detection to ensure results are comparable.

#### 4.6.2 Temporal synchronization of multimodal data

A critical logistical challenge is ensuring temporal synchronization, as the fusion strategy’s validity rests on both modalities capturing the same biological “snapshot” of the tumor. A temporal mismatch between the PET/MRI scan and the blood draw can introduce spurious correlations, particularly under the pressure of active treatment where tumor biology can evolve rapidly. The gold standard for minimizing this biological variance is same-day acquisition, where the blood draw is performed immediately before or after the PET/MRI scan. This approach should be mandated whenever possible in prospective clinical trial protocols. In settings where same-day acquisition is logistically challenging, a pragmatic alternative is to enforce a strict, protocol-defined time window, for example, within ±3 to 7 days. The acceptable duration of this window is context-dependent; a much narrower interval is necessary when monitoring response to fast-acting therapies compared to a baseline assessment in a treatment-naïve patient. Finally, regardless of the chosen synchronization strategy, the meticulous recording of the exact date and time for both the imaging scan and the blood collection is non-negotiable, as this essential metadata allows for rigorous quality control and enables *post-hoc* analyses to assess any potential impact of temporal delays on the results.

#### 4.6.3 Data fusion, sharing, and ethical considerations

The complexity and sensitivity of multimodal data introduce further systemic challenges that span data science, infrastructure, and ethics. From an algorithmic perspective, a core technical bottleneck remains in the multimodal data fusion of high-dimensional image grid data with one-dimensional molecular sequence data, a task that requires the development of novel AI models as discussed in Section 4.5. To train such robust models and validate findings, the field requires large, curated multimodal datasets, making data sharing and interoperability essential. Adopting frameworks such as the FAIR (Findable, Accessible, Interoperable, and Reusable) data principles is necessary to guide the development of shared repositories (91). This process involves establishing common data models and standardized metadata schemas to ensure that imaging data, radiomic features, and genomic variants from different sources can be meaningfully integrated.

However, the richness of these datasets complicates privacy and regulatory compliance. Navigating a complex landscape of regulations, such as the General Data Protection Regulation (GDPR) in Europe and the Health Insurance Portability and Accountability Act (HIPAA) in the US, is mandatory because these combined data are exceptionally personal. De-identification is particularly challenging since imaging may contain unique anatomical details and genomic data is inherently identifiable; in this context, secure federated learning—where models are trained locally without centralizing sensitive data—offers a promising solution. Finally, robust ethical frameworks are indispensable for managing incidental findings from liquid biopsies, such as pathogenic germline mutations or clonal hematopoiesis of indeterminate potential (CHIP). These findings can have profound clinical implications for patients and their families that extend far beyond the scope of the primary oncological investigation, necessitating clear guidelines for disclosure and follow-up.

### 4.7 Health economic and implementation considerations

The substantial cost of both PET/MRI and advanced liquid biopsy presents a formidable barrier to routine clinical adoption. A comprehensive health economic assessment, however, must consider the potential for long-term value derived from avoiding costly, ineffective treatments and enabling earlier, less intensive interventions. This financial challenge is compounded by a difficult reimbursement landscape, where payers typically require high-level evidence of improved patient outcomes before providing coverage, and established codes for such integrated pathways are lacking.

To bridge this gap, a pragmatic path forward is a tiered, risk-stratified implementation model. This strategy would leverage the more accessible liquid biopsy for broader screening or longitudinal monitoring, reserving the high-cost PET/MRI for targeted scenarios, such as localizing disease following a positive ctDNA signal or definitively staging high-risk patients. Such a risk-stratified approach optimizes resource allocation and creates a more economically sustainable pathway toward the clinical integration of this powerful multimodal strategy.

## 5 Conclusions and future perspectives

The deep multimodal data fusion of PET/MRI and liquid biopsy is driving the evolution of cancer diagnosis and treatment toward a multimodal approach. This strategy synergizes macroscopic imaging information with microscopic molecular data, demonstrating clear value in early tumor detection, heterogeneity analysis, dynamic treatment monitoring, and precise prognostic stratification. However, its clinical translation still faces core challenges, including a lack of standardization, algorithmic bottlenecks, and insufficient high-level evidence. Moving forward, leveraging artificial intelligence and multi-omics technologies to build standardized data analysis platforms and validate clinical utility through prospective trials will be essential. The advancement of this integrated paradigm will provide critical technical support for the transition from population-based treatments to individualized precision medicine, ultimately enhancing the systematic and effective management of cancer.

Future directions include: developing AI-driven, multimodal data fusion platforms to achieve end-to-end optimization from raw data to clinical decision-making; exploring the integration of multi-dimensional liquid biopsies beyond blood (such as cerebrospinal fluid and urine) with site-specific imaging for specialized types like central nervous system tumors; building personalized dynamic monitoring networks based on regular liquid biopsies and key time-point PET/MRI scans to enable predictive healthcare; and ultimately forming a closed-loop, integrated diagnostic and therapeutic system. For instance, by leveraging fusion information, patients with high expression of specific targets and carrying sensitive mutations can be identified, enabling simultaneous diagnostic imaging and targeted radionuclide therapy.
